# Fecal Microbiota Transplantation Activity of *Floccularia luteovirens* Polysaccharides and Their Protective Effect on Cyclophosphamide-Induced Immunosuppression and Intestinal Injury in Mice

**DOI:** 10.3390/foods13233881

**Published:** 2024-11-30

**Authors:** He Ma, Abdul Mueed, Yanxu Ma, Muhammad Ibrahim, Ling Su, Qi Wang

**Affiliations:** 1 Engineering Research Center of Chinese Ministry of Education for Edible and Medicinal Fungi, Jilin Agricultural University, Changchun 130118, China; mahetina@163.com (H.M.); ibrahimdagi2017@gmail.com (M.I.); suling0648@jlau.edu.cn (L.S.); 2 College of Plant Protection, Jilin Agricultural University, Changchun 130012, China; 3 State Key Laboratory of Food Science and Technology, Nanchang University, Nanchang 330047, China; amueed3723@yahoo.com; 4 Jilin Sericulture Science Research Institute, Changchun 130012, China; myx187027183@163.com; 5 Tianjin Institute of Industrial Biotechnology, Chinese Academy of Sciences, Tianjin 300308, China

**Keywords:** *Floccularia luteovirens* polysaccharides, fecal microbiota transplantation, immunity and oxidative stress, gut microbiome and metabolites

## Abstract

*Floccularia luteovirens* polysaccharides (FLP1s) have potential biological activities. Our previous study showed that FLP1s positively regulated gut immunity and microbiota. However, it is still unclear whether FLP1s mediate gut microbiota in immunosuppressed mice. This research aims to explore the relationship between FLP1-mediated gut microbes and intestinal immunity in immunosuppressed mice through fecal microbiota transplantation (FMT). The results demonstrated that FLP1s exhibited prebiotic and anti-immunosuppressive effects on CTX-induced immunosuppressed mice. FFLP1 treatment (microbiota transplantation from the fecal sample) remarkably elevated the production of sIgA and secretion of the anti-inflammatory cytokines IL-4, TNF-α, and IFN-γ in the intestine of CTX-treated mice, inducing activation of the MAPK pathway. Moreover, FFLP1s mitigated oxidative stress by activating the Nrf2/Keap1 signaling pathway and strengthened the intestinal barrier function by upregulating the expression level of tight junction proteins (occludin, claudin-1, MUC-2, and ZO-1). Furthermore, FFPL1s restored gut dysbiosis in CTX-treated immunosuppressed mice by increasing the abundance of *Alloprevotella*, *Lachnospiraceae*, and *Bacteroides*. They also modified the composition of fecal metabolites, leading to enhanced regulation of lipolysis in adipocytes, the cGMP-PKG pathway, the Rap1 signaling pathway, and ovarian steroidogenesis, as indicated by KEGG pathway analysis. These findings indicate that FLP1s could modulate the response of the intestinal immune system through regulation of the gut microbiota, thus promoting immune activation in CTX-treated immunosuppressed mice. FLP1s can serve as a natural protective agent against CTX-induced immune injury.

## 1. Introduction

All mammals, including humans, continuously interact with a diverse array of microorganisms inhabiting their intestines throughout their lifespan [1]. The intestine is recognized as a primary habitat for beneficial bacteria where they maintain the properties for their health enhancement. The bacteria living inside the intestine have evolved via natural selection, based on their ability to survive and proliferate in the gut environment [2]. When the intestinal wall becomes densely populated with microorganisms from the intestinal microbiota, a natural defense mechanism is established to prevent direct contact between the intestinal wall and harmful substances [3]. One advantage of this kind of interaction is that alterations inside the microbiota could support or restrain changes in the immune functions of the host and the associated development of immunosuppression illness [4]. Most polysaccharides, including polysaccharides from mushrooms or products of fermentation which reach the intestine through food ingestion, resist stomach acid and bile salts, pass through the upper gastrointestinal tract, replicate, colonize, and play a role within the gut [5]. This procedure could change the composition of intestinal flora and even steer it towards the direction of probiotics, shifting microbiota breeding from being harmful to helpful for holistic health. The gut microbiota do not engage directly with the epithelial cells; instead, the microbiota influence the maturation and function of immune cells indirectly using their metabolic products [6].

Cyclophosphamide (CTX) is widely utilized as a renowned chemotherapeutic agent for the treatment of malignancies and a variety of epithelial tumors [7]. However, the adverse effects of CTX administration on the immune system could result in damage, leading to immunosuppression and risk of leukopenia. Harm to the gastrointestinal mucosa can also trigger alterations in the intestinal microbiota’s composition. In turn, this can result in the migration of harmful bacteria to essential body organs. Where harmful bacteria may migrate can be based on what has commonly been seen in previous experiments of establishing animal models of immunosuppression [8,9]. Maintaining an immunosuppressive state over an extended period of time may lead to various side effects harmful to the body, even causing adverse effects on cardiovascular health [10].

*Floccularia luteovirens* is a well-known wild, edible, and medicinal macro-fungus with a high content of protein and minerals. It grows in the Qinghai–Tibet Plateau and Qilian mountains and is also known as *Armillaria luteovirens* or yellow mushroom, belonging to the genus *Floccularia* [11]. *F. luteovirens* is used as traditional medicine in Tibet for reducing cholesterol levels, releasing neurasthenia, and preventing viral infections [12]. Polysaccharides from *F. luteovirens* could increase the antioxidant ability in PC12 cells to manage oxidative stress induced by H_2_O_2_ and have antiproliferative effects on LO2 and HepG2 cells [13]. The dimeric derived from *F. luteovirens* reduces the growth of tumor cells, including MBL2, L1210, and Hela [14]. The administration of powdered *F. luteovirens* led to a notable improvement in DSS-induced colitis by influencing the NF-κB signaling pathway and recovering the microbiota, leading to an increase in *Lactobacillaceae* and a decrease in *Enterobacteria* [15]. It was also reported that the extracted polysaccharides from an *F. luteovirens* water solution could mitigate renal tissue damage resulting from hyperglycemia [16]. Our previous research demonstrated that *F. luteovirens* polysaccharides (FLP1s) possess gut immunity modulation ability and antioxidant activity through alterations in the gut microbiota and metabolic products [17]. However, whether FLP1-mediated gut microbiota could regulate CTX-induced immunomodulatory activity is still unknown. Therefore, this experiment was conducted to explore the effect of FLP1–fecal microbiota transplantation on intestinal immunity in a CTX-induced model. In this study, a pseudo-germ-free mouse model was used to confirm the intestinal immunomodulatory activity of FLP1s, and then a pseudo-germ-free mouse model and FFLP1 approach were applied to verify the core microbes that might play a key role after FLP1 supplementation. First, we assessed the effect of FFLP1s on intestinal barrier dysfunction and the gut microbiome and metabolome. Secondly, we analyzed the effect of FFLP1s on antioxidant activity, cytokines, and tight junction proteins.

## 2. Materials and Methods

### 2.1. Materials

Purified acidic polysaccharides from *Floccularia luteovirens* (FLP1) with a molecular weight of 21.4 kDa, mainly composed of Glc (68.2%) and GlcA (17.1%), with a small amount of Man (7.5%), Gal (2.9%), and Xyl (4.4%), were obtained from previous research work in our laboratory [17]. CAT (S0051), SOD (S0109), MDA (A003-1), GSH-Px (S0056), and T-AOC (S0119) kits were obtained from Beyotime Biotechnology Co., Ltd. (Shanghai, China). IL-4 (MU30385), IL-6 (MU3044), TNF-α (MU30030), IFN-γ (MU30385), and sIgA (MU30262) ELISA Kits were obtained from Bioswamp Biotechnology Co., Ltd. (Wuhan, China). A BCA protein assay kit was obtained from Life Technologies, Eugene, OR, USA. Protease inhibitors (P6730) and phosphatase inhibitors (P1260) were obtained from Solarbio, Beijing. Other reagents were of analytical grade and commercially available.

### 2.2. Animal Experiment Design

**Figure 1:**
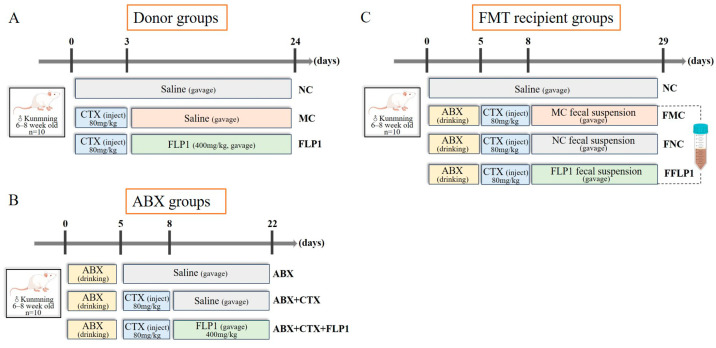
Animal experiments of different groups. (**A**) Donor groups, (**B**) ABX groups, and (**C**) FMT recipient groups.

Male specific pathogen-free (SPF) Kunming mice aged 4 weeks (20 ± 2 g) were supplied by Vital River Laboratory Animal Technology Company (Beijing, China, certificate number: SCXK (Jing) 2021-0006)). After adaptation for 7 days at a temperature of 22 ± 2 °C, humidity of 55 ± 5%, and under a 12/12 h light/dark cycle with free access to food and water, animal experiments were conducted according to the procedures in Figure 1.

Figure 1A shows the donor groups from our previous research work [17]. The FMT samples were collected from the donor group according to the standard protocol [18]. Briefly, a fecal sample was collected from the cecum of each donor group and dissolved in sterile saline until the concentration of the fecal solution was 500 mg/mL, vortexed for 1 min, and centrifuged (1600 rpm) at 4 °C for 5 min. Collected supernatants were filtered through a membrane (70 μm). The obtained supernatants were used as transplantation material. This process was performed under a controlled temperature and time limit to ensure the stability of bacterial composition in the FMT supernatants.

In the pseudo-sterility experiment (Figure 1B), after a week of adaptation, 30 mice were randomly divided into three groups (n = 10) named ABX, ABX + CTX, and ABX + CTX + FLP1. Each group received a gavage with ABX solution containing ampicillin (1 mg/mL), vancomycin (0.5 mg/mL), metronidazole (1 mg/mL), and neomycin (0.5 mg/mL) for 5 days, and cyclophosphamide (CTX) was given by gavage only to the ABX + CTX and ABX + CTX + FLP1 groups for 3 days. Over the following 14 days, the ABX and ABX + CTX groups received 0.9% saline and the ABX + CTX + FLP1 group was administered 400 mg/kg bw/d FLP1.

In the fecal microbiota transplantation (FMT) experiment (Figure 1C), 40 mice were treated with oral administration of ABX mixture for 5 days and then randomly divided into four groups (n = 10). The normal control group (NC) was treated with saline for 29 days. The FMT normal control group (FNC) was injected intraperitoneally with CTX and received a gavage with fecal supernatant of NC donor groups. For FMT treatment with 200 μL per mouse, the CTX model control group (FMC) was injected intraperitoneally with CTX and received a gavage with fecal supernatant of MC donor groups. The FMT FLP1-treated group (FFLP1) was injected intraperitoneally with CTX and received a gavage with fecal supernatant of the FLP1 group in donor groups. The study was conducted according to the guidelines of the Declaration of Helsinki, and all animal treatments were approved by the Animal Ethics Committee of Jilin Agricultural University (20240222001, Changchun, China).

After euthanasia, the spleen thymus, small intestinal, intestinal fluid, and colon fecal matter were collected and stored at −80 °C for further analysis.

### 2.3. Histological Examination

Isolated fresh colon tissues were harvested from the ABX and FMT groups, rinsed with phosphate buffer solution, and preserved in 10% neutral buffered formalin (24 h) for histological examination by embedding in paraffin. Tissues of different mice were sectioned into slices of 5 μm thickness and stained with hematoxylin and eosin (H&E). The tissue slides were then examined for morphological changes with optical microscopy (Nikon, Tokyo, Japan) to analyze the images.

### 2.4. Biochemical Analysis

Intestinal samples (100 mg) were homogenized with pre-cooled PBS (900 µL) and centrifugated at 1500 rpm for 15 min at 4 °C, with the supernatants then collected and stored at −80 °C for further use. The antioxidative activities of superoxide dismutase (SOD), malondialdehyde (MDA), total antioxidant capacity (T-AOC), catalase (CAT), and glutathione peroxidase (GSH-Px) were measured in the small intestines of different groups in triplicate by using commercial assay kits according to the companies’ instructions.

Secretory Immunoglobulin A (sIgA), interleukin-4 (IL-4), interleukin-6 (IL-6), interferon-γ (INF-γ), and tumor necrosis factor-alpha (TNF-α) levels in the intestinal samples were measured with commercial ELISA kits according to the instructions of the manufacturer.

### 2.5. Western Blot Assay

Intestinal tissues (small intestine) were homogenized with 10-time RIPA lysis buffer (Beyotime, Shanghai, China) containing 1% protease inhibitor and 0.8% phosphatase inhibitors. The lysate was centrifuged at 12,000× *g* for 15 min at 4 °C, and supernatants were collected for further measurement. The protein concentration was identified using a bicinchoninic acid (BCA) assay kit. After denaturation, a protein sample of 30 µg was separated using sodium dodecyl sulfate–polyacrylamide gel electrophoresis (SDS-PAGE) and transferred to polyvinylidene difluoride (PVDF) membranes. Then, the membranes were blocked with 5% skim milk in the shaker for 60 min. After washing three times with Tris-buffered saline Tween-20 (TBST), the samples were incubated with primary antibodies (Cell Signaling Technology, Boston, MA, USA) overnight at 4 °C. After further washes, they were incubated with matching secondary antibodies (Millipore, Billerica, MA, USA) and conjugated with horseradish peroxidase for 2 h at room temperature. Finally, the immunoreactive bands were visualized by an enhanced chemiluminescence (ECL) system (Bio-Rad, Hercules, CA, USA). The relative band intensity was quantified with Image J 1.53 software (National Institutes of Health, USA). Antibody information of Western Blot determination is shown in Supplementary Table S1.

### 2.6. Gut Microbiota Analysis

After 29 days of FMT treatment, the cecum contents of the mice were frozen in liquid nitrogen and stored at −80 °C. Total DNA was extracted and purified, and 16S ribosomal RNA gene microbiome sequencing analysis was performed by Biomarker Technologies Co., Ltd. (Beijing, China). The hypervariable region V3-V4 of the bacterial 16S rRNA was amplified with the primers 338F (5′-ACTCCTACGGGAGGCAGCAG-3′) and 806R (5′-GACTACHVGGGTWTCTAAT-3′). PCR products were checked and purified using an Omega DNA purification kit (Omega Inc., Norcross, GA, USA). Purified amplification products were sequenced using the Illumina MiSeq platform (Illumina, San Diego, CA, USA) according to the standard protocols [19]. Bioinformatics analysis of this study was performed using BMKCloud (www.biocloud.net, accessed on 20 May 2024).

### 2.7. Fecal Metabolomics Analysis

Untargeted metabolomics was used to quantify feces metabolites, performed by Biomarker Technologies Co., Ltd. (Beijing, China). All experimental groups were assessed with 5 biological replicates. The preparation of the samples, the conditions of HPLC-MS/MS, the method, and data analysis were carried out according to the previous method [17].

### 2.8. Statistical Analysis

All the experiments were conducted at least in triplicate, and all statistical analyses were performed using SPSS 22.0 and GraphPad Prism 8.0 software. Origin 10.5 software was used to process data graphically. The values are presented as mean ± standard deviation (SD). Differences between groups were compared using analysis of variance (ANOVA) with Duncan’s multiple comparisons tests, with a critical *p*-value < 0.05 considered statistically significant and represented by different letters. Correlations between microbiota biomarkers and different metabolites were determined using Spearman correlation analysis.

## 3. Results

### 3.1. Effects of FLP1 on CTX-Induced Immunosuppression and Intestinal Injury in Mice

**Figure 2:**
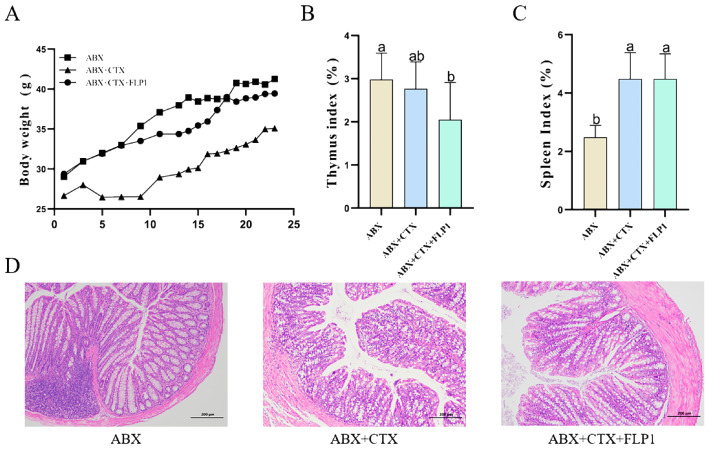
The influence of FLP1 gavage in ABX groups. (**A**) Body weight. (**B**) Thymus index. (**C**) Spleen index. (**D**) H&E staining of colon sections for different groups (scale bar = 200 μm). Data are presented as mean ± SD (n = 6). Different letters over bars denote the statistical significance between two groups (*p* < 0.05).

To check whether the gut microbiota plays an important role in FLP1 ameliorating CTX-induced immunosuppression and intestinal damage, the ABX solution was used to eliminate intestinal flora in mice for the pseudo-sterile experiment. As shown in Figure 2A, the body weight reduction of the mice was significantly recovered in the ABX group and ABX + CTX + FLP1 group compared to the ABX + CTX group. Interestingly, the ABX + CTX + FLP1 group recovered better than the ABX + CTX group, which is mainly due to the function of FLP1. The thymus and spleen are the fundamental immune organs that play an important role in immunoregulation, which reflects the immunity function. After CTX injection, the immune organ indices of the mice were dramatically reduced (Figure 2B,C). The thymus index was significantly (*p* < 0.05) increased in the ABX group compared to the ABX + CTX group. Interestingly, the thymus index was significantly (*p* < 0.05) reduced in the ABX + CTX + FLP1 group compared to the ABX group and ABX + CTX group. Similarly, the spleen index was significantly (*p* < 0.05) increased in the ABX + CTX + FLP1 group compared to the ABX group, while no significant difference in spleen index was found between the ABX + CTX + FLP1 group and ABX + CTX group. This phenomenon indicated that FLP1 ameliorated CTX-induced immunosuppression in mice through the gut microbiota.

A healthy intestinal epithelium with mucosal integrity is essential for preventing the translocation of bacteria and endotoxins within the intestine, which helps avoid inflammatory responses and the spread of oxidative stress to other circulatory systems or tissues [20]. Figure 2D illustrates that the ABX + CTX group displayed pronounced pathological characteristics of intestinal damage compared to the ABX group. The intestinal epithelial cells were extremely damaged, the integrity of the intestinal mucosal membrane was disrupted, the quantity of goblet cells was significantly reduced, and the length of villi was shortened in the ABX + CTX group. After FLP1 intervention, the H&E stains revealed obvious improvement effects on the intestinal damage caused by CTX treatment, especially in intestinal mucosa integrity. Our results suggest that FLP1 can amend Cy-induced intestinal damage.

### 3.2. Effects of FLP1 on CTX-Induced Oxidative Stress and Intestine Immune Dysfunction

**Figure 3:**
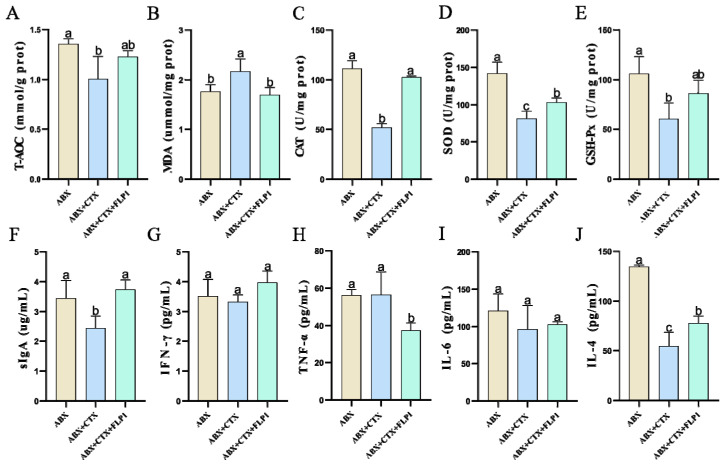
The regulated secretion of immunomodulatory and antioxidant effects in ABX groups. (**A**–**E**) Small intestinal oxidative enzyme (T-AOC, MDA, CAT, SOD, and GSH-Px) levels in mice. (**F**–**J**) Small intestinal cytokine (sIgA, IFN-γ, TNF-α, IL-6, and IL-4) levels in mice. Data are presented as mean ± SD (n = 6). Different letters over bars denote the statistical significance between two groups (*p* < 0.05).

Oxidative stress is tightly interlinked to immunoecology. CTX could induce oxidative stress in the body, thereby reducing the levels of antioxidant enzymes. The results shown in Figure 3A–E illustrate that the CAT and SOD levels were significantly (*p* < 0.05) increased in the ABX + CTX + FLP1 group compared to the ABX + CTX group. In contrast, the MDA level was significantly (*p* < 0.05) decreased in the ABX + CTX + FLP1 group compared to the ABX + CTX group, while no significant difference in T-AOC and GSH-Px levels was observed between the ABX + CTX + FLP1 group and the ABX + CTX group. Interestingly, the SOD level was significantly (*p* < 0.05) increased in the ABX + CTX + FLP1 group compared to the ABX group.

Immunity-related cytokines and immunoglobulins were measured in colon tissues to further assess the effect of FLP1 on colon mucosal immunity. The results shown in Figure 3F–J demonstrate that the levels of sIgA and IL-4 were significantly (*p* < 0.05) increased in the ABX + CTX + FLP1 group compared to the ABX + CTX group. In contrast, the level of TNF-α was significantly (*p* < 0.05) decreased in the ABX + CTX + FLP1 group, while no significant difference in IFN-γ and IL-6 levels was observed between the ABX + CTX + FLP1 group and the ABX + CTX group. Interestingly, the TNF-α and IL-4 levels were significantly (*p* < 0.05) increased in the ABX + CTX + FLP1 group compared to the ABX group, while no significant difference in IL-6 and IFN-γ levels was observed between the ABX + CTX + FLP1 group and the ABX group. These results imply that FLP1 intervention can alleviate CTX-induced oxidative stress and immune dysfunction in pseudo-sterile experiments.

### 3.3. Effects of FFLP1 on CTX-Induced Immunosuppression and Intestinal Injury

*F. luteovirens* polysaccharides (FLP1s) have been reported to ameliorate Cy-induced intestinal microbiota disorders, and the hypothesis that intestinal microbiota play a critical role in FLP1’s ability to attenuate CTX-induced immunosuppression and intestinal injury was assessed. To verify our hypothesis, 16S rDNA gene sequencing was performed on the cecal contents of the FNC group, the FMC group, and the FFLP1 group, with fecal microbiota transplanted from NC, MC, and FLP1.

**Figure 4:**
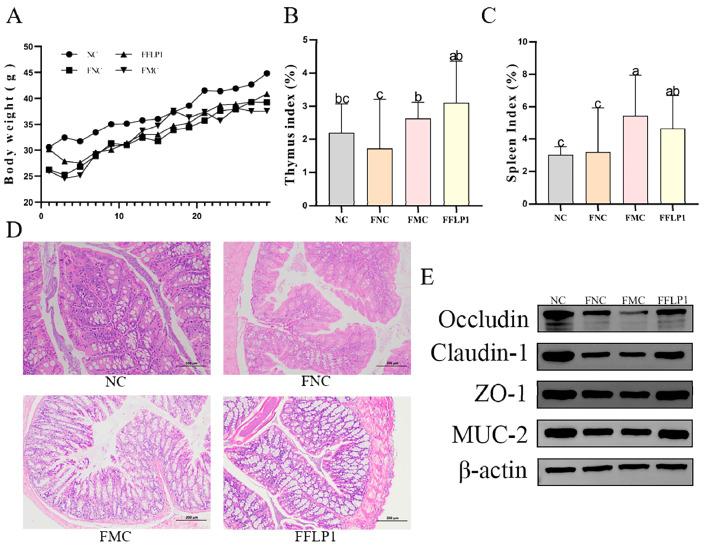
The influence of FFLP1 in immunosuppressed mice. (**A**) Body weight. (**B**) Thymus index. (**C**) Spleen index. (**D**) H&E staining for colon tissues in different groups (scale bar = 200 μm). (**E**) Bands of occludin, claudin, ZO-1, and MUC-2 expression in the intestinal mucosa, with β-actin used as a standard. Data are presented as mean ± SD (n = 6). Different letters over bars denote the statistical significance between two groups (*p* < 0.05).

ABX was administrated to the mice for 5 days to eliminate gut microbiota, and the weights of the NC, FNC, FMC, and FFLP1 groups were reduced, indicating that the intestinal flora plays an important role during mouse growth. Figure 4A shows that the body weight was significantly decreased in the FMC group compared to the FNC group. After the FFPL1 intervention, the body weight significantly increased compared to the FMC and FNC groups. Moreover, Figure 4B,C illustrate that the thymus and spleen indices were significantly increased in the FMC group compared to the NC group. In contrast, there was no significant difference found in the thymus index between the FMC and FFLP1 groups, while the spleen index was significantly (*p* < 0.05) increased in the FFLP1 group compared to the FMC group. This suggests that the FFLP1 from donor groups could attenuate the CTX-induced dysfunction.

Further H&E staining of colon tissue was analyzed to investigate the effect of FFPL1 on CTX-induced intestinal injury. Figure 4D shows that deformation of colonic crypt tissues, loss of goblet cells, and damage of mucous membrane were found more in the FMC group compared to the NC and FNC groups. After FFLP1 administration, the mice significantly recovered from the CTX-induced deformation of colonic crypt tissues, loss of goblet cells, and damage of mucous membrane compared to the FMC group.

The intestinal barrier is crucial in stopping the movement of bacteria and other byproducts, functioning as an important defense in maintaining the homeostasis of the internal environment [21]. The experiment further included a Western blot experiment to analyze the hypothesis that FMT administration has positive effects in the function of protecting intestinal mucosa damaged by CTX treatment. The expressions of Muc-2, ZO-1, occludin, and claudin-1 (Figure 4E) were pronouncedly reduced in the FMC group after receiving CTX injections, while FFLP1 significantly (*p* < 0.05) upregulated the expression of Muc-2, ZO-1, occludin, and claudin-1, which further emphasizes the potential role of FFLP1 in protecting the integrity of the intestinal barrier.

### 3.4. Effects of FMT on Oxidative Enzyme Secretion in CTX-Treated Groups

**Figure 5:**
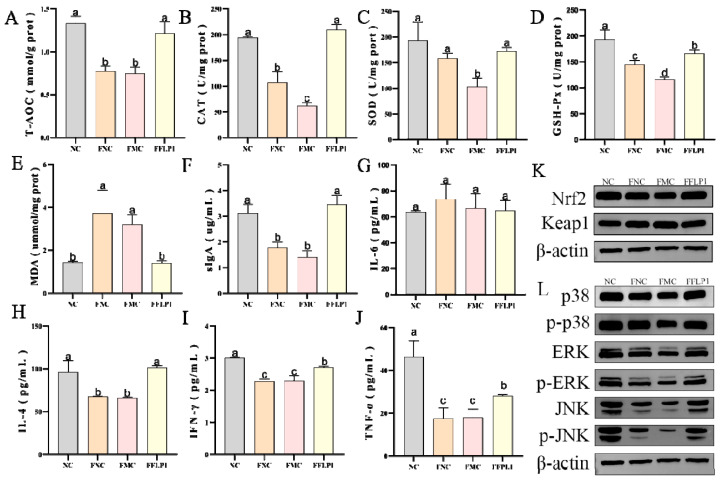
The regulated secretion of immunomodulatory and antioxidant effects in FMT groups. (**A**–**E**) Small intestinal oxidative enzyme (T-AOC, CAT, SOD, GSH-Px, and MDA) levels in mice. (**F**–**J**) Small intestinal cytokine (sIgA, IL-6, IL-4, IFN-γ, and TNF-α) levels in mice. (**K**) Bands of Nrf2 and Keap1 expression, with β-actin used as a standard. (**L**) Bands of p-38, p-p38, ERK, p-ERK, JNK, and p-JNK in intestinal mucosa, with β-actin used as a standard. Data are presented as mean ± SD (n = 6). Different letters over bars denote the statistical significance between two groups (*p* < 0.05).

CTX is recognized for generating free radicals within the body and leading to oxidative stress [22]. To investigate the effects of FFLP1 on oxidative stress in immunosuppressed mice, the levels of the oxidative enzymes T-AOC, CAT, SOD, and GSH-Px were detected. Figure 5A–D illustrate that the T-AOC, CAT, SOD, and GSH-Px levels were significantly decreased in the FMC group compared to the FNC group. After FFLP1 intervention, the levels of T-AOC, CAT, SOD, and GSH-Px were significantly increased compared to the FMC and FNC groups (*p* < 0.05). In contrast, the MDA level (Figure 5E) was significantly downregulated in the FFLP1 group compared with the FMC and FNC groups (*p* < 0.05).

Moreover, the Nrf2/keap1 protein level was evaluated to examine its involvement in ROS removal through FFLP1 intervention (Figure 5K). The Nrf2 expression exhibited a significantly lower level in the FMC group compared to the NC group, while after FFLP1 administration, Nrf2 expression was remarkably improved, which showed a clear significant difference compared to the FMC group (*p* < 0.05). However, the upregulated level of Keap1 in the FMC group was notably reversed by the treatment of FFLP1 (*p* < 0.05). These results suggest that FFLP1 can alleviate the CTX-induced intestinal oxidative stress in immunosuppressed mice.

### 3.5. Effects of FMT on Intestine Immune Dysfunction Induced by CTX

The immunoglobulin and immune cytokine results (Figure 5F–J) show that sIgA, IL-4, IFN-γ, and TNF-α levels were significantly decreased in the FMC group after the CTX injection. FFLP1 intervention significantly increased the levels of sIgA, IL-4, IFN-γ, and TNF-α compared with the FMC and FNC groups (*p* < 0.05). Interestingly, no significant difference was found in sIgA and IL-4 levels between the FFLP1 and NC groups. In contrast, no statistical difference in IL-6 levels between the NC, FNC, FMC, and FFLP1 groups was found.

To confirm the effect of FFLP1 administration on immunomodulatory activity in CTX-induced immunosuppressed mice, the MAPK protein analysis was further used, including p-38, JNK, and ERK. The expression levels of p-p38/p-38, p-JNK/JNK, and p-ERK/ERK (Figure 5L) were remarkably decreased in the FMC group compared to the NC group (*p* < 0.05). After the administration of FFLP1, the relative protein expression of p38, JNK, and ERK protein phosphorylation levels significantly recovered (*p* < 0.05). Therefore, it was affirmed that FFLP1 enhanced the intestinal barrier function by reinstating the immune-related proteins.

### 3.6. FMT Regulation of Intestinal Microbiota

**Figure 6:**
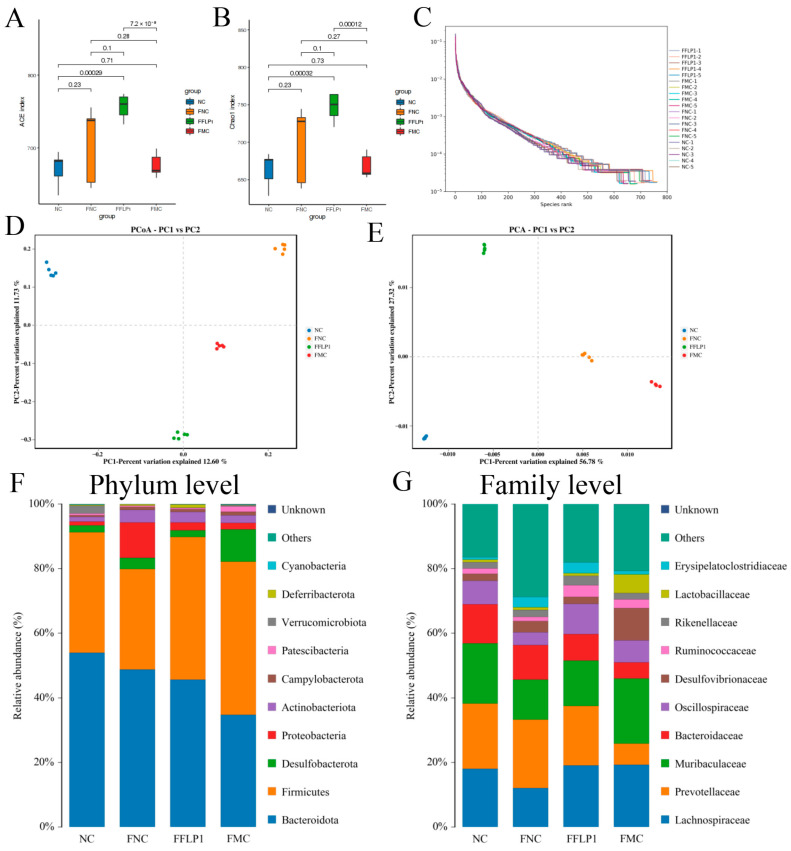
Regulation of gut microbiota of immunosuppressed mice by FMT treatment. (**A**) ACE index, (**B**) Chao1 index, and (**C**) rank–abundance curves for all samples. (**D**) PCoA analysis and (**E**) PCA analysis of each sample’s microbial composition. Community bar plot at the (**F**) phylum level and (**G**) family level. Data are presented with n = 5.

Further, 16S rRNA analysis was performed to determine whether FFLP1 can alleviate CTX-induced immunosuppression through the gut microbiota. The *α*-diversity of the gut microbiota is shown in Figure 6A,B. ACE and Chao 1 indices were significantly decreased in the FMC group compared to the FNC group, indicating that CTX administration significantly reduced the *α*-diversity of the gut microbiota. After administration of FFLP1, the indices of *α*-diversity were significantly increased (*p* < 0.05). Furthermore, significant differences in the Simpson and Shannon diversity indices were observed between the FFLP1 group and the FMC group (Supplementary Figure S1). The β-diversity was further appraised via the principal coordinate analysis (PCoA) of Bray–Curtis and principal component analysis (PCA). As shown in Figure 6D, the FFLP1 groups were clustered in the same region, with separation from the FMC group cluster along axis 2 (PCo2) with a high proportion of variation. The PCA showed that the groups were divided into four different clusters, which indicated a conspicuous separation in bacterial structures among the NC, FNC, FMC and FFLP1 groups (Figure 6E). These results suggest that the diversity of the intestinal flora was recovered by FFLP1 administration.

To identify the different taxa, the relative abundances at the level of phylum and family were further assessed. The top 10 phyla showed that the relative abundance of *Bacteroidetes* was decreased in the FMC group compared to the FNC and NC groups. Similarly, the relative abundance of *Firmicutes* was increased in the FMC group compared to the FNC and NC groups. After the FFLP1 intervention, an increased relative abundance of *Bacteroidetes* and a decreased relative abundance of *Firmicutes* in the FFLP1 group compared to the FMC group were noted. The relative abundance of *Desulfobacterota*, *Campylobacteria*, and *Proteobacteria* was increased in the FMC group compared to the FNC group and the NC group. The abundances of these microbiota decreased after the FFLP1 intervention. Similarly, FFLP1 administration increased the abundance of *Actinobacteriota* and *Verrucomicrobiota* in CTX-induced immunosuppressed mice (Figure 6F). Further, the top 10 family genera showed that the relative abundance of *Prevotellaceae*, *Oscillospiraceae*, and *Bacteroidaceae* was dramatically decreased in the FMC group compared to the FNC and NC groups, indicating that CTX intervention induced gut dysbiosis (Figure 6G). In addition, the genus-level analysis showed that FFLP1 administration increased the abundance of *Alloprevotella* and decreased the abundance of *Desulfovibrio* compared to the FMC group (Supplementary Figure S2).

**Figure 7:**
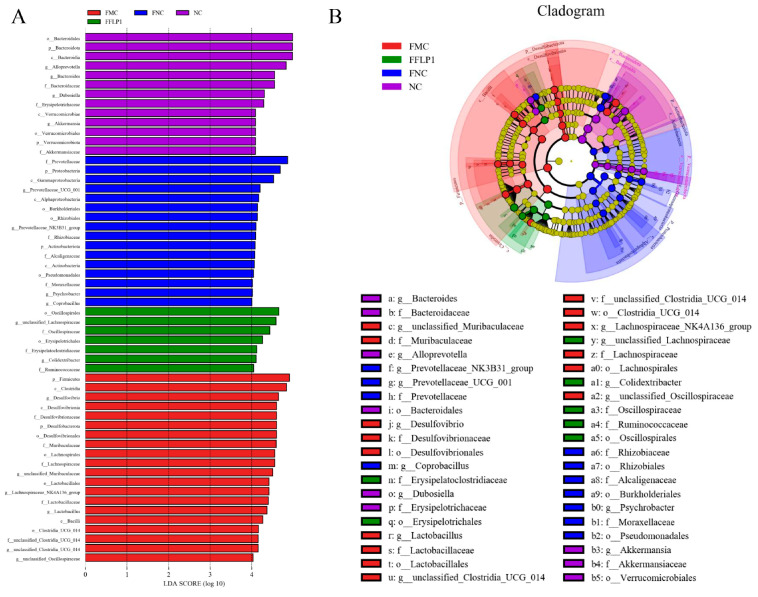
FMT treatment modulated the composition of gut microbiota in immunosuppressed mice. (**A**) Histogram of the LDA scores. (**B**) Bar chart of LEfSe analysis. Data are presented with n = 5.

Furthermore, LEfSe analysis was employed to distinguish the differences in microbial composition between the NC, FNC, FMC, and FFLP1 groups (Figure 7A). Our findings revealed a total of 56 distinct bacterial species among the four groups (LDA > 4, *p*-value < 0.05). *Oscillospirales*, *unclassified_Lachnospiraceae*, and *Oscillospiraceae* were abundant in the FFLP1 group, while *Firmicutes*, *Clostridia*, and *Desulfovibrio* were abundant in the FMC group. The FNC group had an abundance of *Prevotellaceae*, *Proteobacteria*, and *Gammaproteobacteria*. The results of the cladogram analysis shown in Figure 7B are consistent with the LEfSe results.

### 3.7. FMT Regulation of Metabolites in Fecal Sample

**Figure 8:**
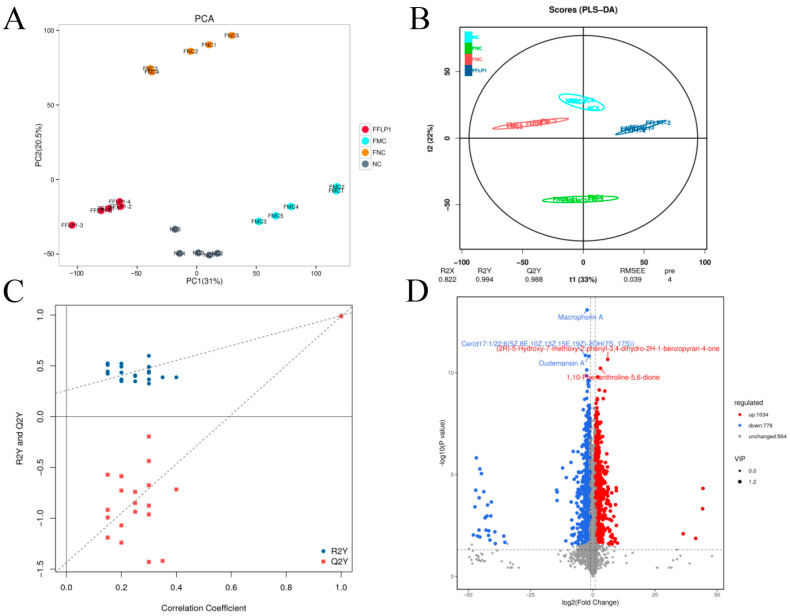
FMT treatment alters the level of fecal metabolites in Cy-treated immunosuppressed mice. (**A**) Score plot of the PCA model. (**B**) Score plot of the PSL-DA model. (**C**) Permutations test of the PSL-DA model. (**D**) Volcano plot. Data are presented with n = 5.

**Figure 9:**
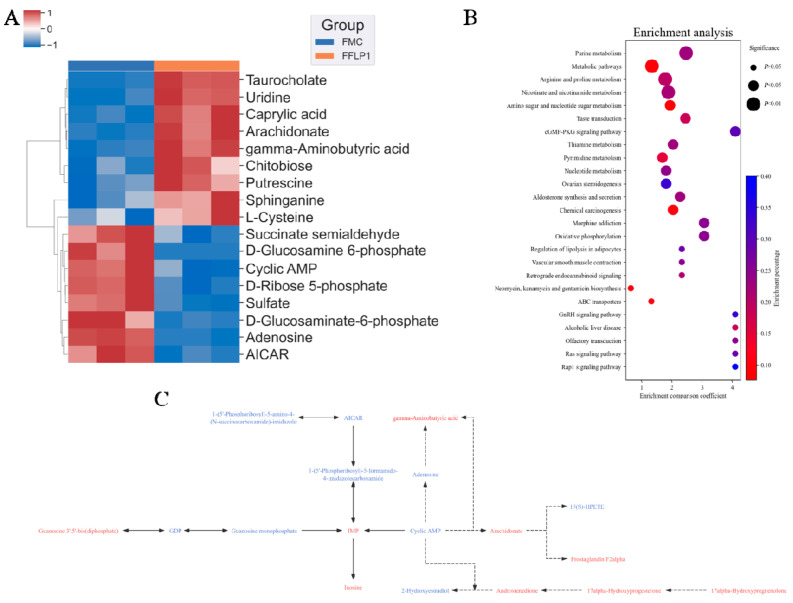
(**A**) Heatmap of different metabolites in FFLP1 and FMC groups. (**B**) Metabolomic pathway enrichment analysis. (**C**) Metabolic pathway analysis of cyclic AMP; red indicates an increase in metabolite levels, blue indicates a decrease, solid lines represent direct interactions, dashed lines represent indirect interactions, single arrows indicate unidirectional regulation, and double arrows indicate bidirectional regulation. Data are presented with n = 5.

Non-targeted metabolomics analysis was used to examine the fecal metabolomic profiles in the NC, FNC, FMC and FFLP1 groups. A total of 6159 metabolites were identified from twenty samples of four groups (n = 5). A principal component analysis (PCA) and partial least squares discriminant analysis (PLS-DA) were performed, and the model score plots are shown in Figure 8A,B. All samples of the four groups were classified into four separate clusters, which indicated that there were noticeable variances among the NC, FNC, FMC, and FFLP1 groups. The permutations test was employed to verify the reliability of the PLS-DA (Figure 8C) and showed that the slope of the fitted regression line was positive, demonstrating that the model was valid and independent without overfitting. A volcano plot was further used to identify the differences of metabolites. Figure 8D shows that based on the criteria of variable importance for projection (VIP) > 1.2, *p*-value < 0.05, and log_2_ (fold change) < 0.5 or >2, a total of 1034 potential metabolites were upregulated, while 779 were downregulated between the FMC and FFLP1 groups. Heatmap analysis of the differential metabolites between FFLP1 and FMC showed that FFLP1 primarily increased the levels of arachidonate, chitobiose, and taurocholate while decreasing the levels of uridine and cyclic AMP (Figure 9A). A metabolic pathway enrichment analysis was performed to further understand the metabolic alterations. The KEGG pathway analysis showed that FFLP1 intervention increased the regulation of lipolysis in adipocytes, the cGMP-PKG pathway, the Rap1 signaling pathway, and ovarian steroidogenesis (Figure 9B). In the KEGG database analysis of all pathways involving cyclic AMP, the relevant metabolites were analyzed, and the results are shown in Figure 9C. As cyclic AMP levels decreased, the levels of guanosine 3′,5′-bis(diphosphate), gamma-aminobutyric acid, arachidonate, prostaglandin F2alpha, and inosine increased, while 2-hydroxyestradiol and adenosine levels decreased. Additionally, sulfate, D-ribose 5-phosphate, adenosine, cyclic AMP, and AICAR were associated with purine metabolism; putrescine, S-adenosyl-L-methionine, L-ornithine, and L-proline were associated with arginine and proline metabolism; and succinate semialdehyde, gamma-aminobutyric acid, and NAD+ were associated with nicotinate and nicotinamide metabolism (Supplementary Table S2).

### 3.8. Correlation Between Gut Microbiota, Metabolites and Biomarkers

**Figure 10:**
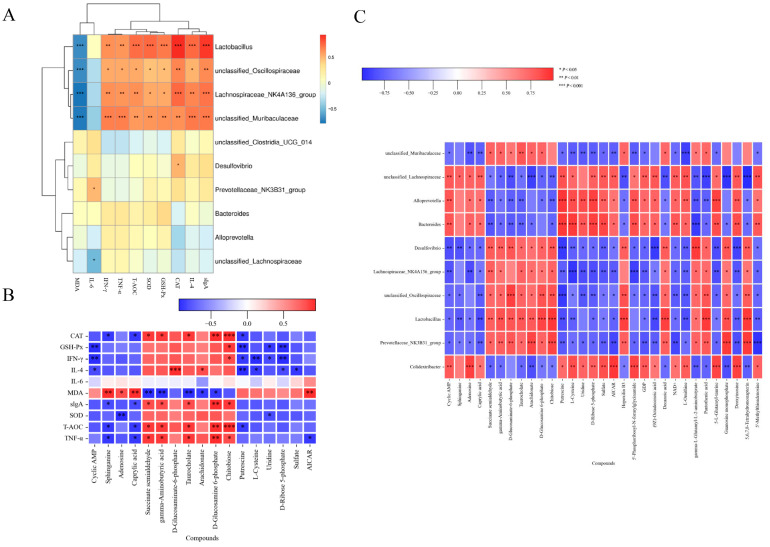
Correlation between different gut microbiota, gut metabolites, and biomarkers. (**A**) Correlation between different gut microbiota and biomarkers related to immunity. (**B**) Correlation between different gut metabolites and biomarkers related to immunity. (**C**) Correlation between different gut microbiota and metabolites. Spearman analysis was used for the matrix (* *p* < 0.05, ** *p* < 0.01, *** *p* < 0.001).

The underlying mechanism of FFLP1’s effect on immunosuppression was further investigated by examining the correlation between gut microbiota, fecal metabolites, antioxidant indices, and immunity markers. Figure 10A shows that *Lactobacillus*, *Oscillospiraceae*, *Muribaculaceae*, and *Lachnospiraceae_NK4A136_group* were significantly positively correlated with sIgA, IL-4, CAT, GSH-Px, SOD, T-AOC, and TNF-α and significantly negative correlated with MDA, while having no correlation with IL-6. *Lachnospiraceae* had a negative correlation with IL-6. The results suggest that the treatment of FFLP1 led to changes in the gut microbiota and in antioxidant and immunity biomarkers, which are essential factors in managing immunosuppressive diseases, and a certain internal connection exists between them.

Additionally, the correlation between gut metabolites and immunosuppression markers (Figure 10B) showed that cyclic AMP and putrescine had a significant negative correlation with GSH-Px, IFN-γ, and IL-4, while succinate semialdehyde, gamma-aminobutyric acid, and taurocholate significantly positively correlated with CAT, sIgA, T-AOC, and TNF-α and significantly negatively correlated with MDA. Putrescine, L-cysteine, uridine, and D-ribose 5-phosphate had a significant negative correlation with IFN-γ and IL-6.

Furthermore, the correlation between gut microbes and metabolites is shown in Figure 10C. The results show that *Muribaculaceae*, *Lachnospiraceae_NK4A136_group*, *Oscillospiraceae*, *Lactobacillus*, and *Prevotellaceae_NK3B31_group* showed a significant negative correlation with cyclic AMP, adenosine, and caprylic acid, while *Lachnospiraceae*, *Alloprevotella*, *Bacteroides*, and *Colidextribacter* showed a significantly negative correlation with cyclic AMP, adenosine, and caprylic acid. Similarly, *Lachnospiraceae*, *Alloprevotella*, *Bactteroides*, and *Colidextribacte* had a significant negative correlation with L-cysteine, uridine, sulfate, and putrescine but a significant positive correlation with decanoic acid, pantothenic acid, and 5-L-glutamyl-turine. These correlations suggest that gut microbiota (*Lachnospiraceae*, *Alloprevotella*, and *Bacteroides*) and gut metabolites (cyclic AMP, D-glucosaminate-6-phosphate, and D-glucosamine-6-phosphate) play a significant role in CTX-induced immunosuppressed mice.

## 4. Discussion

*F. luteovirens*, often referred to as *Armillaria luteovirens*, is an endemic fungus commonly found in the Qinghai–Tibet Plateau and the Qilian Mountains that is well known in China as a medicinal and edible macro-fungus [12]. It helps to ease numbness, dizziness, headaches, insomnia, and nervous exhaustion [14]. Recent research on *F. luteovirens* has shown that it could protect against lung cancer [23], regulate immunomodulatory activity [14], and relieve intestinal inflammation [24]. Our previous study proved that purified polysaccharides from *F. luteovirens* (FLP1s) could enhance antioxidative abilities and improve immune regulation by changing intestinal flora; additionally, changes in metabolites due to FLP1s were studied through metabolomics research. Therefore, the present study aimed to further elucidate the mechanism by which FLP1s improve immune function and intestinal injury in immunosuppressed mice through an immunosuppressive donor assay, FMT assay, and pseudo-sterility assay. Several researchers have indicated that the therapeutic benefits of FMT treatment for intestinal injury are mainly observed in the colon [25,26], because the transplanted fecal microorganisms likely primarily colonize the colon [27]. Therefore, it is hypothesized that FFLP1 reaches the colon and is utilized by intestinal microorganisms.

Pseudo-germ-free mouse models are widely used to study intestinal microbiota. The mice were allowed to freely consume antibiotic solutions to deplete the majority of their gut microbiota, subsequently establishing an immunosuppression model for further experimental evaluation. The results showed that there was no significant difference between the ABX + CTX and ABX + CTX + FLP1 groups in terms of body weight changes, immune organ index, and physiological and biochemical indices, while compared with the ABX group, both groups had significant differences (Figure 2 and Figure 3). This phenomenon was consistent with the fact that polysaccharides from carboxymethylated sources prevent CTX-induced immunosuppression and intestinal injury. Based on our current findings, FLP1s can ameliorate CTX-induced immunosuppression and intestinal injury in an intestinal microbiota-dependent manner.

CTX damage to the intestinal barrier triggers the release of reactive oxygen species (ROS) and other oxidative substances disrupting the intracellular redox balance. This oxidative stress response further exacerbates intestinal damage, creating a vicious cycle that weakens the immune defense of the organism [28,29]. A recent study showed that *P. ostreatus* polysaccharides attenuate CTX-induced immune intestinal oxidative stress [30]. In the present study, CTX disrupted the equilibrium balance between the antioxidant and oxidative systems. However, this imbalance was reversed after the FFLP1 intervention. This was demonstrated by a marked increase in antioxidant enzymes, including SOD, CAT, T-AOC, and GSH-Px, alongside a significant reduction in MAD, thereby alleviating CTX-induced oxidative damage to colonic tissues (Figure 5).

Secretory immunoglobulin A (sIgA) is the predominant antibody, belonging to the immunoglobulin family, produced by plasma cells in the mucous lamina propria, which is the primary substance utilized by the gastrointestinal tract to inhibit bacterial invasion [31,32]. It also regulates the activities of bacteria and formulates the composition of the gut microbiota [33]. Additionally, cytokine secretion is essential for intercellular communication, which possesses an immunomodulatory capacity. IL-2, IFN-γ, and TNF- α are primarily involved in cellular immune responses [34,35], while IL-4 and IL-6 are primarily involved in humoral immune responses [36,37]. Under normal conditions, cytokine levels maintain a dynamic balance to support normal cellular and humoral immune functions. A previous study demonstrated that a 200 mg/kg dose of *A. auricula* polysaccharides (AAP1) significantly elevated TNF-a, IL-2, IL-4, and IFN-γ levels in CTX-induced immunosuppressed mice, thereby improving immune function [38]. Our findings revealed a significant reduction in cytokines and immunoglobulin levels in the colon of mice after CTX injection. However, FLP1 remarkably enhanced the secretion of IL-4 and sIgA, while it had no significant effect on IFN-γ, TNF-α, and IL-6. Hence, FLP1 may possess even stronger immunomodulatory activity through gut microbiota. However, how these microbiota and metabolites exert their therapeutic effects and the specific molecular mechanisms by which FLP1 alleviates immunosuppression and intestinal injury are still unclear.

The intestinal microbiota serve as a crucial link between nutrition and human health, influencing various physiological aspects including host nutrition, disease development, drug metabolism, and regulation of the immune system [39]. Intestinal microbiota imbalance is an important environmental factor that induces intestinal barrier dysfunction [40], affecting local mucosal immune homeostasis and potentially impacting systemic immune responses via lymphatic and systemic circulation [41]. Fecal microbiota transplantation (FMT) is a method of transplanting feces from a healthy donor into the intestine of another patient to reverse intestinal microbiota dysbiosis in the recipient [42]. In addition, FMT has been used to differentiate between causal and non-causal effects of specific intestinal microbiota in the therapeutic effects of drugs/diets [43]. Therefore, in an attempt to explore the role of intestinal microbiota in FLP1’s ability to alleviate CTX-induced immunosuppression and intestinal injury, mice were pseudo-sterile treated with NC as a blank control, and FMC and FFLP1 were transplanted from the microbiota of the MC and FLP1 groups (optimal dose for therapeutic effect), respectively, for the subsequent experimental assays.

The FMT experiment was conducted to explore the effect of FFLP1 on CTX-induced immune intestinal injured mice through the gut microbiome and metabolome. FMT offers an innovative therapy for metabolic diseases by reshaping the gut microbiota as it transfers stool from a healthy donor to the recipient to restore gut microbiota balance [44]. FMT has been proven effective when used in the therapy of inflammatory bowel disease [45], ulcerative colitis [46], and type 2 diabetes [47]. In this study, FFLP1 demonstrated a potent immunomodulatory effect, significantly mitigating CTX-induced weight loss, spleen and thymus atrophy, as well as splenic and colonic lesions (Figure 4). Additionally, FFLP1 alleviated CTX-induced oxidative stress, immunosuppression, and disruption of the intestinal barrier. Overall, the FFLP1 group exhibited a substantial increase in the level of SOD, T-AOC, CAT, GSH-Px, IL-4, IFN-γ, TNF-α, and slgA in the colon, along with a marked reduction in MDA content compared to the FMC group. Nrf2 is the primary cell sensor for oxidative stress, which is bound to Keap1. During oxidative stress, Keap1’s active site is oxidized, inhibiting its interaction with Nrf2; this kind of inhibition triggers the movement of Nrf2 into the nucleus [48]. After this movement, Nrf2 connects with the antioxidant response element, which results in oxidative stress [49]. It has been reported that lentinan could prevent the increase in ROS and the production of and decrease in Nrf-2 and HO-1 levels triggered by LPS while inhibiting the NF-κB and MAPK pathways [50]. FMT in rats treated with Saikosaponin A could alleviate severe acute pancreatitis by activating the Keap1/Nrf2-ARE antioxidant signaling pathway [51]. Our study is consistent with the finding that FFLP1 significantly increased Nrf2 protein levels while decreasing Keap1 protein levels. Our results also suggest that FFLP1 protects against immunosuppressive activity in mice by reducing oxidative stress through the activation of the Nrf2/Keap1 signaling pathway.

Furthermore, dysregulated expression of tight junction proteins (occludin, claudin-1), mucoproteins (MUC-2), and scaffolding proteins (ZO-1) on the intestinal epithelium can significantly impair intestinal barrier integrity. Recently, studies have shown that ZO-1 and occludin are involved in regulating epithelial cell apoptosis and proliferation, controlling viral and bacterial entry, and coordinating specialized epithelial structure [52]. Claudin-1 is a critical transmembrane protein that plays a pivotal role in maintaining the structural integrity, barrier function, and permeability of the intestine [53]. In this study, FFLP1 counteracted the CTX-induced downregulation of ZO-1, occludin, claudin-1, and MUC-2 protein expression (Figure 4). Therefore, this study hypothesized that FFLP1 could promote the immune response by restoring mucosal mechanical barrier damage through upregulation of tight junction proteins in CTX-induced immunosuppressed mice. Intestinal defense against infections and maintenance of its integrity depend on the continuous activation of MAPK-mediated signaling pathways in some respects [54]. The p38, JNK, and ERK MAPK signaling pathways are reported to be upstream regulators of IL-6 and TNF-α [55]. It has been reported that baicalin-derived FMT could restore intestinal injury via the MAPK signaling pathway, which involves the MAP3K11, MAP4K2, MAPK7, and MAPK13 genes [56]. In this study, our results indicated that FFLP1 could increase the phosphorylated proteins of p38, JNK, and ERK from the MAPK pathway, thereby enhancing the CTX-induced intestinal dysfunction.

Furthermore, the 16S rRNA results showed that FFLP1 modulated the CTX-induced microbiota disorder, making the microbiota composition similar to the NC group. Nevertheless, FFLP1 significantly upregulated the abundance of the beneficial genera *Bacteroidetes*, *Actinobacteriota*, *Verrucomicrobiota Prevotellaceae*, and *Oscillospiraceae* and significantly downregulated the abundance of *Desulfobacterota*, *Campylobacteria*, and *Proteobacteria*. As nonpathogenic microorganisms, probiotics such as *Bifidobacterium*, *Lactobacillus*, and other beneficial microbiota play a crucial role in enhancing both intestinal and systematic immunity [57,58]. These microorganisms are essential components of the intestinal flora, contributing positively to the host’s immune function. It has been recognized that *Prevotellacea* enhances host intestinal immunity by producing butyric acid [59]. FFLP1 treatment increased the abundance of *Prevotellaceae* compared to the FMC group. It has been reported that the effectiveness of FMT treatment in ulcerative colitis patients is related to the modulation of *Prevotellaceae* [59], aligning with this study’s trend. A previous study reported that FMT treatment increased the abundance of *Alloprevotella*, which might have offered therapeutic potential for ulcerative colitis by modulating bile acid metabolism, enhancing the intestinal barrier, and regulating immune responses [60]. The results showed that FFLP1 treatment recovered the abundance of *Alloprevotella* in CTX-induced immunosuppressed mice. Prototypical genera of *Oscillospiraceae* can produce short-chain fatty acids, such as butyric acid, that have beneficial effects on the host [61]. In this study, FFLP1 treatment increased the abundance of *Oscillospiraceae* members belonging to *Firmicutes*, which could enhance the gut barrier and modulate the immune system, and also increased *Bacteroidaceae*, which are essential for the digestion of complex carbohydrates and protection against pathogenic bacteria [62,63]. *Desulfovibrio* represents a controversial group of bacteria that can promote health and contribute to disease [64]. Its abundance in the intestine is modulated by factors such as diet and probiotics [64]. Studies have shown that *Desulfovibrio* produces short-chain fatty acids, predominantly acetic acid, which play a critical role in supporting host intestinal health [65]. *Actinobacteria* phyla are disease-causing organisms that typically cause localized inflammation and septicemia [66]. Our results showed that FFLP1 treatment decreased the abundance of *Desulfovibrio* and *Actinobacteria*. In conclusion, FFLP1 may ameliorate immunosuppression and intestinal injury by modulating intestinal microbiota.

After FMT treatment, various metabolites were identified, with a significant proportion primarily involved in nucleotide metabolism, including purine metabolism, pyrimidine metabolism, and arginine and proline metabolism, which belongs to amino acid metabolism. Purine metabolism is pivotal in maintaining the balance of immune responses, especially in regulating inflammation and immune cell activity [67]. It is also linked to gut microbiota, which could modulate purine metabolism by producing or degrading purine metabolites such as adenosine and xanthine, then interacting with immune cells and promoting anti-inflammatory effects or triggering pro-inflammatory responses [68]. As a precursor for glutathione synthesis, L-cysteine exhibits potent antioxidant properties [69], suggesting that FFLP1 may protect cells from oxidative stress by modulating glutathione metabolism. *Lactobacilli* facilitate gut mucosal development by converting arginine into L-ornithine, which subsequently boosts the production of L-kynurenine, a ligand for the aryl hydrocarbon receptor, through tryptophan metabolism in gut epithelial cells [70]. Arginine and proline metabolism is crucial for immune and gut microbiota balance. Arginine promotes immune cell activity by producing nitric oxide, while polyamines such as putrescine support immune regulation, particularly during inflammation [71]. The amino sugar and nucleotide sugar metabolism pathway was significantly enriched between the FFLP1 and FMC groups, which plays a crucial role in preserving intestinal mucosal barrier integrity and antimicrobial peptide production [72]. Under these metabolic pathways, multiple immune-related metabolites were identified such as adenosine, cyclic AMP, guanosine monophosphate, uridine, CMP, MP, UDP, cytosine, and deoxycytidine, which belong to nucleosides and nucleotides, as well as L-cysteine, L-ornithine, L-proline, S-adenosyl-L-methionine, putrescine, N-carbamoyl putrescine, and sarcosine, which belong to amino acids and derivatives. Cyclic AMP (cAMP) acts as a secondary messenger, negatively regulates the MAPK pathway through complex mechanisms, and primarily involves cAMP-dependent protein kinase A (PKN), which could inhibit the activation of Ras and subsequently reduce downstream MAPK signaling [73].

In the KEGG database analysis of all pathways involving the key metabolite cyclic AMP, it was observed that 2-hydroxyestradiol exhibits anti-inflammatory properties, and a decrease in its expression may lead to a relative increase in pro-inflammatory signals, which can further stimulate immune cell activation, potentially aiding in overcoming immunosuppression [74]. Arachidonate is the precursor of the pro-inflammatory molecules prostaglandin and leukotriene, which are usually associated with immune activation. However, excessive levels of arachidonate may trigger excessive inflammatory responses, leading to tissue damage [75,76]. While the levels of arachidonate and prostaglandin F2alpha raised, the level of gamma-aminobutyric acid (GABA) also increased. GABA could inhibit potential excessive inflammatory responses, prevent uncontrolled inflammation, and avoid damage to the body; it also interacts with the enteric nervous system to enhance the integrity of the intestinal barrier and reduce oxidative stress-induced damage to intestinal cells [77,78,79]. Guanosine 3′,5′-bis(diphosphate) plays a crucial role in the cellular stress response. At elevated levels, it helps enhance immune cell activity and overcome the immunosuppressive state [80]. Inosine monophosphate (IMP) is an important intermediate in purine metabolism whose increase indicates the enhancement of purine metabolism, which helps restore the proliferation and activity of immune cells in immunosuppressive models, thereby boosting disease resistance [81]. Inosine possesses anti-inflammatory and immunomodulatory properties, and increases in its level help inhibit excessive inflammatory responses and regulate the immune system and are beneficial for restoring immune balance and reducing CTX-induced excessive immune stress [82,83]. FFLP1 dynamically maintains immune homeostasis by regulating cyclic AMP and its interacting metabolites, which helps enhance immune function and maintain overall balance. Considering the results of the Western blot showing that FFLP1 activated the MAPK pathway and the reduced cyclic AMP level detected in the FFLP1 group, it is speculated that the polysaccharide FLP1 displays its immune effect through gut microbiota-dependent activation of the MAPK pathway for intestinal immunity.

## 5. Conclusions

This research offers a comprehensive insight into FLP1’s immunomodulatory effects and provides evidence that FLP1 attenuates Cy-induced immune dysfunction and intestinal injury in mice. Overall, the beneficial effects of FFLP1 on CTX-induced immunosuppression and intestinal injury in mice appear to be related to changes in the composition of the intestinal microbiota, which in turn influence changes in metabolites. These findings present an excellent rationale for FLP1 to be an effective tactic to ameliorate Cy-induced immune injury.

## Data Availability

The original contributions presented in the study are included in the article; further inquiries can be directed to the corresponding author.

## Supplementary Materials

The following supporting information can be downloaded at: https://www.mdpi.com/article/10.3390/foods13233881/s1, Figure S1: Shannon and Simpson indices; Figure S2. Relative abundance of gut microbiota in FMT groups at genus level; Table S1: Antibody information of Western blot determination; Table S2: Significantly changed pathways and metabolites involved in FFLP1 and FMC groups.
