# Supplementary material for: Fecal Microbiota Transplantation Activity of Floccularia luteovirens Polysaccharides and Their Protective Effect on Cyclophosphamide-Induced Immunosuppression and Intestinal Injury in Mice
Source: Foods. 2024 Nov 30;13(23):3881. doi: 10.3390/foods13233881 (PMC11640258; doi:10.3390/foods13233881)
Supplement: Supplementary file 1 [file foods-13-03881-s001.zip › foods-3292551-supplementary.pdf]

Probiotic properties of Floccularia luteovirens Polysaccharides and its protective effect on Cyclophosphamide-induced immunosuppression and intestinal injury in mice: A fecal microbiota transplantation study

He Ma[1,2] †, Abdul Mueed[3] †, Yanxu Ma[4], Muhammad Ibrahim[1,2], Ling Su[1,2] *, Qi Wang[1,2,*]

[1] Engineering Research Center of Chinese Ministry of Education for Edible and Medicinal Fungi, Jilin Agricultural University, Changchun 130118, China (H.M) mahetina@163.com: (M.I) ibrahimdagi2017@gmail.com

[2] College of Plant Protection, Jilin Agricultural University, Changchun 130012, China (L.S) suling0648@jlau.edu.cn

[3] State Key Laboratory of Food Science and Technology, Nanchang University, Nanchang, Jiangxi, China (A.M) amueed3723@yahoo.com

[4] Jilin sericulture Science Research Institute, Changchun 130012, China (Y.M) 187027183@qq.com

† Equal contribution

* Correspondence: qiwang@jlau.edu.cn

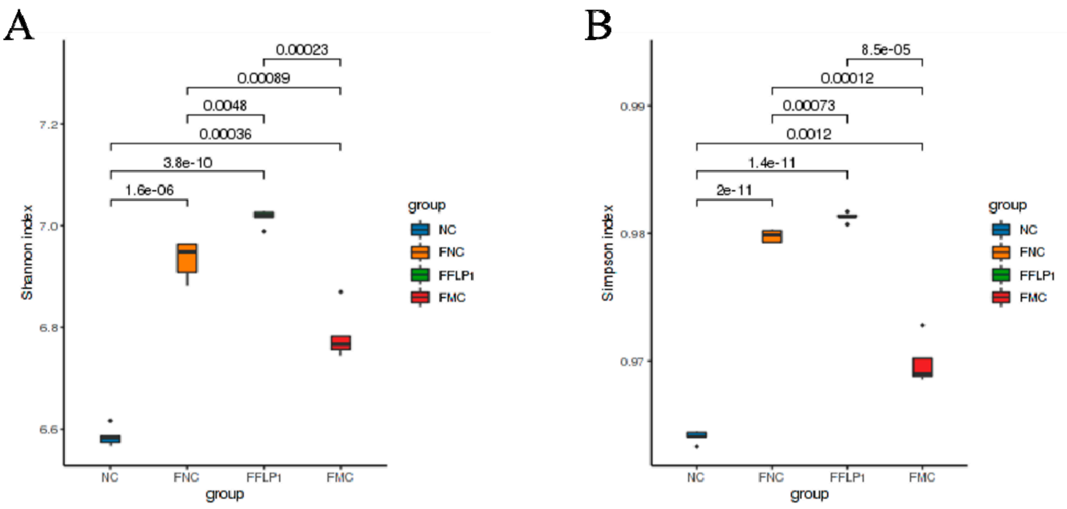

Figure S1 Shannon and Simpson index

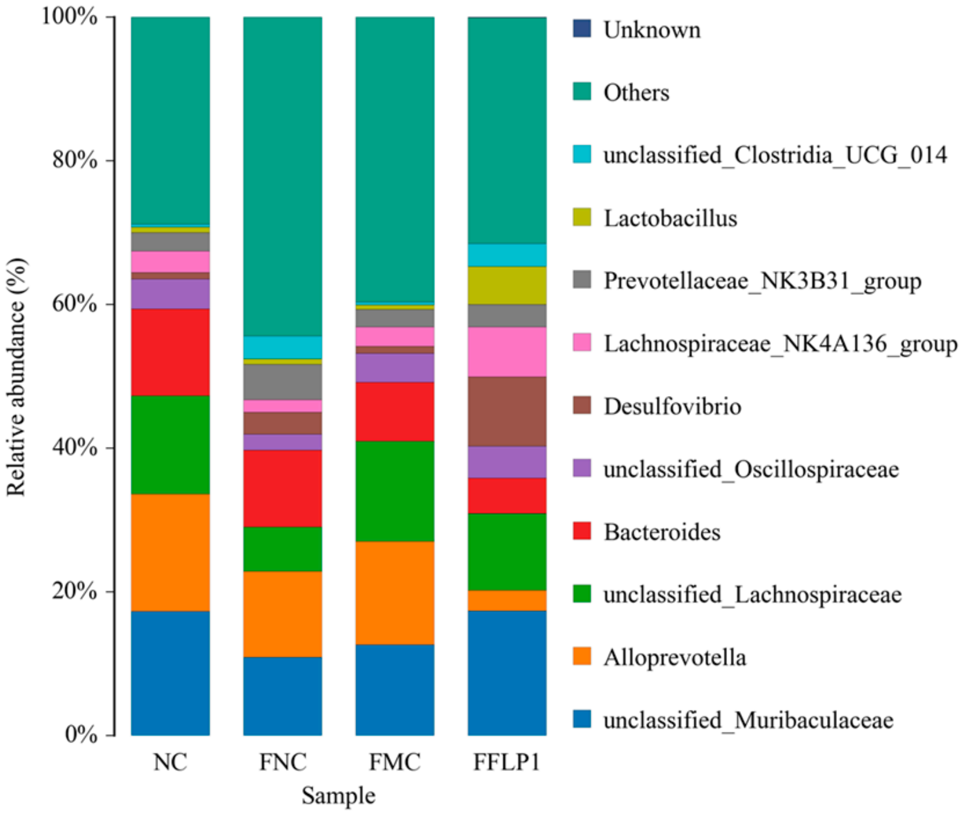

Figure S2. Relative abundance of gut microbiota in FMT groups at genus level

Table S1. Antibody information of Western Blot determination

Antibody	Dilution ratio	Article number	Company
Occludin	1:5000	27260-1-AP	Proteintech Group
Claudin-1	1:3000	28674-1-AP	Proteintech Group
MUC-2	1:2000	27675-1-AP	Proteintech Group
ZO-1	1:5000	21773-1-AP	Proteintech Group
Nrf2	1:2000	16396-1-AP	Proteintech Group
Keap1	1:2000	10503-2-AP	Proteintech Group
ERK	1:8000	11257-1-AP	Proteintech Group
p-ERK	1:1000	AF1891	Beyotime
p38	1:1000	AM065	Beyotime
p-p38	1:1000	D3F9	Cell Signaling Technology
JNK	1:1000	AF1048	Beyotime
P-JNK	1:1000	AF1762	Beyotime

Table S2. Significantly changed pathway and metabolites involved in FFLP1 and FMC groups

Pathway name	P-value	VIP	Compounds	Compound name
Purine metabolism	0.00007	1.267	C00059, C00117, C00212, C00575, C04677	Sulfate, D-Ribose 5-phosphate, Adenosine, Cyclic AMP, AICAR
Metabolic pathways	0.00128	1.225	C00117, C00334, C00232, C04677, C00035, C00836, C05122, C00134, C00097, C00352	D-Ribose 5-phosphate, gamma-Aminobutyric acid, Succinate semialdehyde, AICAR, Sphinganine, Taurocholate, Putrescine, L-Cysteine, D-Glucosamine 6-phosphate
Arginine and proline metabolism	0.00108	1.228	C00134, C00019, C00077, C00148, C00213, C00436	Putrescine, S-Adenosyl-L-methionine, L-Ornithine, L-Proline, Sarcosine, N-Carbamoyl putrescine
Nicotinate and nicotinamide metabolism	0.00026	1.265	C00232, C00334, C00003, C00006, C00153	Succinate semialdehyde, gamma-Aminobutyric acid, NAD+, NADP+, Niacinamide
Amino sugar and nucleotide sugar metabolism	0.00037	1.199	C00352, C01674, C04037	D-Glucosamine 6-phosphate, Chitobiose, 1-Phospho-alpha-D-galacturo nate
cGMP-PKG signaling pathway	0.00013	1.268	C00212, C00575, C00144	Adenosine, Cyclic AMP, Guanosine monophosphate
Thiamine metabolism	0.00069	1.207	C00097, C02892	L-Cysteine, Thiamine acetic acid
Pyrimidine metabolism	0.00001	1.268	C00299, C00015, C00055 C00380, C00881	Uridine, UDP, CMP, Cytosine, Deoxycytidine
Nucleotide metabolism	0.00015	1.271	C00212, C00130, C00144,	Adenosine, IMP, Guanosine monophosphate

Notes: The P-values and VIP values represent the means of compounds.
